# Association of preoperative CT-derived visceral adipose tissue index with synchronous metastasis and metastasis-free survival after curative-intent surgery in colorectal cancer

**DOI:** 10.3389/fonc.2026.1830599

**Published:** 2026-06-02

**Authors:** Ke Yin, Yuchen Xie, Qingling Li, Guanyi Liao, Song He, Jinjun Guo

**Affiliations:** 1 Department of Radiology, Bishan Hospital of Chongqing Medical University, Chongqing, China; 2 Chongqing Key Laboratory of Precision Medicine for Digestive System Tumors, Bishan Hospital of Chongqing Medical University, Chongqing, China; 3 Department of Gastroenterology, Bishan Hospital of Chongqing Medical University, Chongqing, China; 4 Department of Gastroenterology, The Second Affiliated Hospital of Chongqing Medical University, Chongqing, China; 5 College of Basic Medicine, Chongqing Medical University, Chongqing, China

**Keywords:** body composition, colorectal cancer, computed tomography, metastasis-free survival, synchronous metastasis, visceral adipose tissue index

## Abstract

**Background:**

To evaluate whether computed tomography (CT)-derived visceral adipose tissue index (VATI) is associated with synchronous metastasis and metastasis-free survival (MFS) after curative-intent surgery in colorectal cancer (CRC).

**Methods:**

This retrospective two-center study included patients with newly diagnosed CRC who underwent abdominal CT within 1 month before diagnosis or initial treatment between January 2019 and December 2022 (n = 468; metastatic CRC [mCRC], n = 77; non-metastatic CRC [nmCRC], n = 391). For the synchronous metastasis analysis, mCRC and nmCRC were propensity score matched 1:1 for age, sex, carcinoembryonic antigen (CEA), carbohydrate antigen 19-9 (CA19-9), study center, and tumor location (77 pairs), and multivariable logistic regression was performed. For the MFS analysis, the surgical nmCRC cohort was stratified by sex-specific median VATI into low-VATI (n = 195) and high-VATI (n = 196) groups, and Kaplan-Meier and Cox proportional hazards analyses were performed. Among patients who developed postoperative metastasis (n = 96), an exploratory descriptive comparison was performed between early (≤ 2 years, n = 76) and late (> 2 years, n = 20) metastasis groups.

**Results:**

After matching, mCRC showed higher subcutaneous adipose tissue index (SATI), VATI, and visceral-to-subcutaneous fat area ratio (VSR) than nmCRC. VATI was independently associated with synchronous metastasis (OR, 1.110; 95% CI, 1.067–1.155; p < 0.001), whereas SATI and VSR were not. In the surgical nmCRC cohort, the high-VATI group had shorter MFS (log-rank p = 0.001). On multivariable Cox analysis, VATI remained independently associated with shorter MFS (HR, 1.017; 95% CI, 1.003-1.032; p = 0.021), together with CA19-9, CEA, N stage, and lymphovascular invasion (LVI). In the exploratory descriptive comparison, no statistically significant differences in body composition metrics were observed between the early and late metastasis groups.

**Conclusion:**

Preoperative CT-derived VATI is a reproducible opportunistic imaging biomarker associated with synchronous metastasis and shorter MFS after curative-intent surgery and may help refine risk stratification and postoperative surveillance in CRC, although prospective validation is required before routine clinical application.

## 1 Introduction

Colorectal cancer (CRC) remains one of the most common malignancies worldwide, with a substantial global burden in incidence and mortality (1–3). Distant metastasis is a major cause of treatment failure and death, and contemporary clinical practice guidelines repeatedly emphasize optimal management and surveillance strategies for metastatic CRC (mCRC) (4, 5). Both patients who present with synchronous metastasis at diagnosis and those who develop distant metastasis after curative-intent surgery require complex treatment planning and intensive follow-up (6). Therefore, a reproducible and readily available biomarker derived from routine preoperative assessments could be clinically valuable for identifying patients at high risk of metastasis and tailoring postoperative surveillance.

Obesity is associated with CRC development and adverse outcomes; however, body mass index (BMI) reflects overall body size and cannot capture heterogeneity in fat distribution or metabolic risk. Accumulating evidence suggests that visceral adipose tissue (VAT) accumulation, which reflects insulin resistance, adipokine imbalance, and chronic low-grade inflammation, may promote invasion and metastasis through the tumor-host axis (7, 8). Recent radiomics and deep learning studies further indicate that combining tumor imaging features with visceral fat phenotypes may help identify occult peritoneal dissemination risk (9). Visceral obesity has also been linked to perioperative complications and recovery after CRC surgery (10). CT-based body composition analysis enables opportunistic, quantitative assessment without additional imaging burden and may provide a practical foundation for clinical risk stratification (11, 12). With advances in automated and semantic segmentation networks, CT-derived body composition parameters may be integrated into routine imaging workflows (13).

Beyond energy storage, VAT functions as an active endocrine and immune organ. Translational studies indicate that adipose stromal cells and adipocyte-derived exosomes can remodel the tumor microenvironment via factors such as interleukin-6 and hepatocyte growth factor, thereby contributing to invasion, dissemination, and therapy resistance (14, 15). Nevertheless, systematic multicenter evidence linking preoperative CT-quantified visceral fat burden to synchronous metastasis and postoperative metastasis risk in CRC remains limited. Accordingly, we quantified the visceral adipose tissue index (VATI) on preoperative CT and investigated its associations with synchronous metastasis, early versus late postoperative metastasis, and metastasis-free survival (MFS).

## 2 Materials and methods

### 2.1 Study design and participants

**Figure 1:**
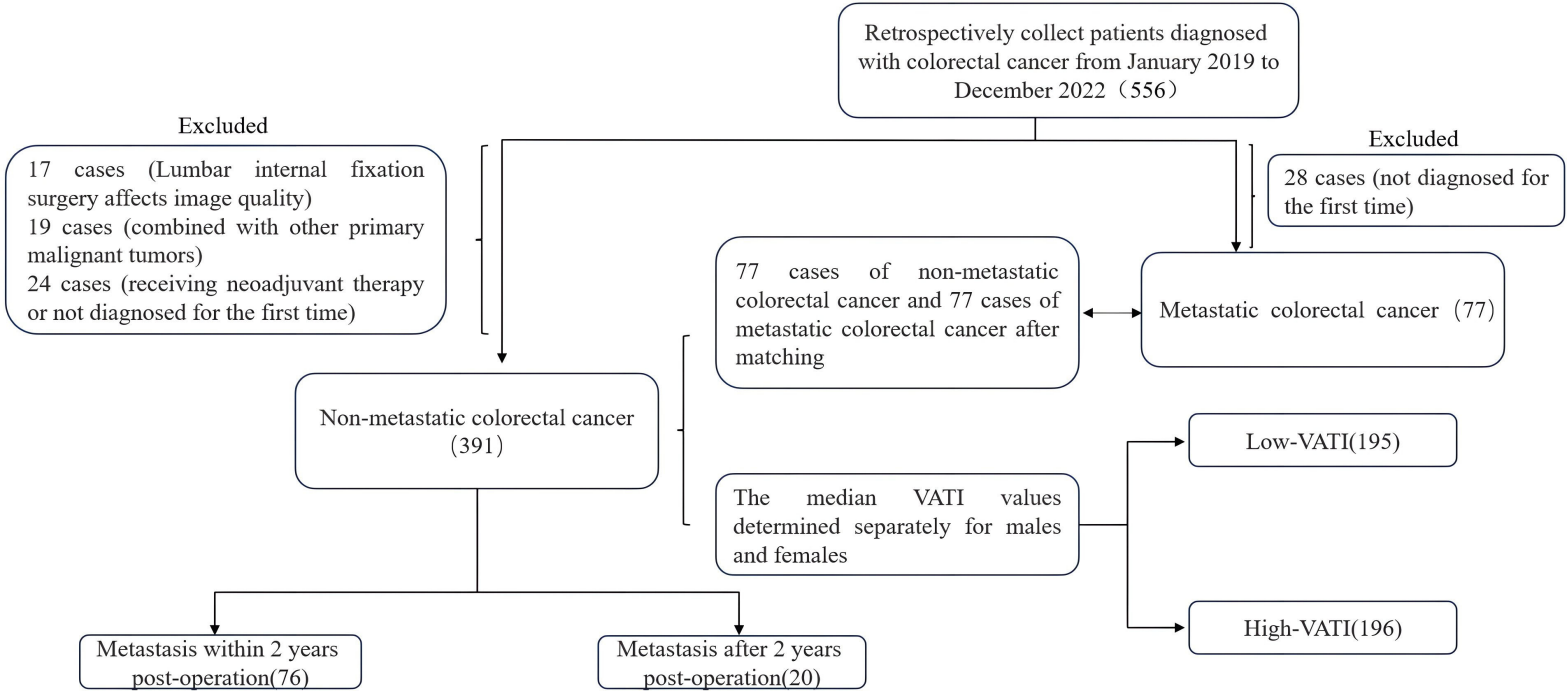
Patient selection and cohort construction. CA19-9, carbohydrate antigen 19-9; CEA, carcinoembryonic antigen; LVI, lymphovascular invasion; PNI, perineural invasion; VATI, visceral adipose tissue index.

This retrospective two-center cohort study reviewed patients with newly diagnosed CRC who underwent abdominal CT within 1 month before diagnosis or treatment between January 2019 and December 2022. The institutional Ethics Review Committees of both centers approved this study and waived the requirement for informed consent (approval No. cqbyklyy-20240918-24), and all data were anonymized. A total of 556 patients were screened; 28 were excluded because they were not newly diagnosed, leaving 77 patients with mCRC and 451 with non-metastatic CRC (nmCRC). In the nmCRC group, 60 additional patients were excluded due to lumbar internal fixation compromising image quality (n = 17), concomitant other primary malignancies (n = 19), or receipt of neoadjuvant therapy or not being newly diagnosed (n = 24). The final study population comprised 468 patients (mCRC, n = 77; nmCRC, n = 391) (**Figure 1**). Inclusion criteria were: (a) pathologically confirmed CRC, (b) adequate CT image quality for body composition analysis, and (c) complete clinical data (at minimum, sex, age, height and weight, CEA, and CA19-9; and pathologic data for patients who underwent curative surgery). Exclusion criteria were: (a) not newly diagnosed, (b) concurrent malignancy or major organ failure, and (c) severe abdominal surgical history or artifacts that precluded reliable segmentation. This study adhered to the Strengthening the Reporting of Observational Studies in Epidemiology (STROBE) statement (16).

### 2.2 Cohort construction

Three analytic cohorts were constructed (**Figure 1**). First, for the synchronous metastasis analysis, patients with mCRC and nmCRC were matched 1:1 using nearest-neighbor propensity score matching (caliper = 0.2) based on pretreatment baseline variables that were uniformly available across groups, including age, sex, CEA, CA19-9, study center, and tumor location, yielding 77 matched pairs. Second, for the exploratory early versus late postoperative metastasis analysis, among patients with nmCRC who underwent curative-intent surgery and subsequently developed distant metastasis, patients were classified as early metastasis (≤ 2 years after surgery; n = 76) or late metastasis (> 2 years after surgery; n = 20). Third, for the MFS analysis, the surgical nmCRC cohort (n = 391) was stratified into low-VATI (n = 195) and high-VATI (n = 196) groups using sex-specific median VATI values.

### 2.3 CT-derived body composition and clinical variables

**Figure 2:**
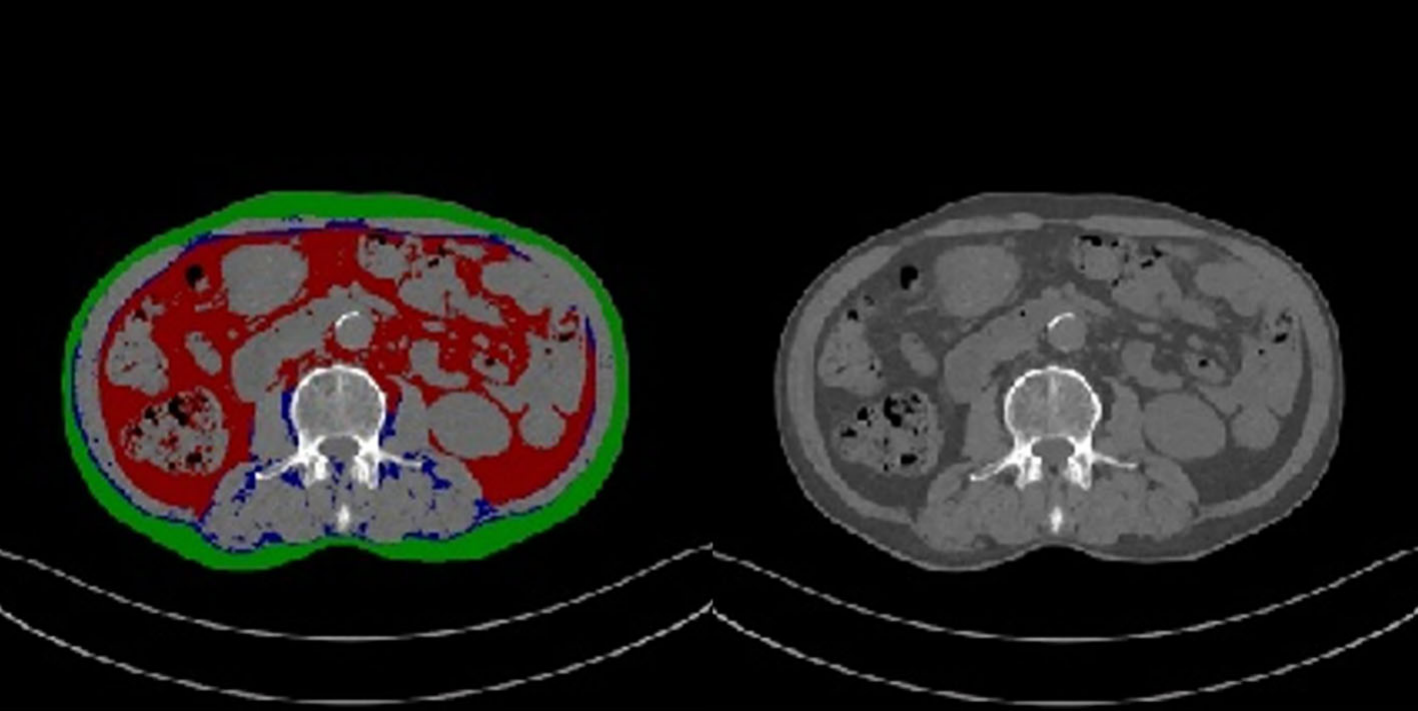
Example of CT-based body composition segmentation at the L3 level. Green, subcutaneous adipose tissue area; red, visceral adipose tissue area; blue, intermuscular adipose tissue area.

CT examinations were performed using GE Revolution CT and Siemens SOMATOM Definition AS scanners with the following parameters: 120 kV, automatic tube current modulation, 512 x 512 matrix, and 5-mm slice thickness/interval. Body composition segmentation was performed on a single axial slice at the mid-L3 level using semi-automated software (Bones QCT, Bones Technology Limited, Hong Kong, China). Adipose tissue compartments were segmented using established Hounsfield unit thresholds: visceral and intermuscular adipose tissue, -150 to -30 HU; subcutaneous adipose tissue, -190 to -50 HU (17, 18) (**Figure 2**). Cross-sectional areas were indexed by height squared to derive the intermuscular adipose tissue index (IMATI), VATI, and subcutaneous adipose tissue index (SATI). The visceral-to-subcutaneous fat area ratio (VSR) was calculated as visceral fat area divided by subcutaneous fat area. Two trained readers performed measurements blinded to outcomes and jointly reviewed segmentations; boundaries were corrected by consensus when required. To assess interobserver reproducibility, a subset of 50 cases was independently measured by both readers. Interobserver agreement for VATI, SATI, and IMATI was quantified using the intraclass correlation coefficient (ICC) with 95% confidence intervals. CEA and CA19–9 were dichotomized using clinically relevant thresholds (CEA, 5 ng/mL; CA19-9, 37 U/mL), and BMI was dichotomized at 25 kg/m^2^. Single-slice L3 CT assessment has been widely validated to represent whole-body muscle and fat burden (11, 19).

### 2.4 Statistical analysis

Continuous variables are presented as mean ± standard deviation or median (interquartile range, IQR), as appropriate, and were compared using the Student t test or Mann-Whitney U test. Categorical variables are presented as counts (percentages) and were compared using the chi-squared test or Fisher exact test. In the synchronous metastasis analysis, covariate balance before and after matching was assessed using standardized mean differences (SMDs), with an absolute SMD < 0.10 considered acceptable balance. In the matched cohort, multivariable logistic regression was performed to evaluate the association between VATI and synchronous metastasis, and odds ratios (ORs) with 95% confidence intervals (CIs) are reported. To improve clinical interpretability, VATI-associated effect estimates were additionally reported per one IQR increase in both the synchronous metastasis analysis and the MFS analysis. For the exploratory early versus late postoperative metastasis analysis, patients with postoperative distant metastasis were stratified according to time to metastasis (≤2 years vs >2 years), and body composition, clinical, and pathologic variables were compared directly between groups using univariable methods. For the postoperative survival analysis, MFS was defined as the interval from the date of curative-intent surgery to the date of first documented distant metastasis. Patients without documented distant metastasis, including those who died without documented metastasis, were censored at the date of last follow-up. Kaplan-Meier curves were compared using the log-rank test. Univariable and multivariable Cox proportional hazards regression analyses were performed to identify factors associated with MFS. Variables with p < 0.10 in univariable analysis and variables considered clinically relevant were entered into the multivariable Cox model. Because this was a two-center study, a sensitivity Cox model was additionally fitted with study center forced into the multivariable model to assess whether the association between VATI and MFS was robust to center-related heterogeneity. The proportional hazards assumption was assessed using Schoenfeld residuals. All tests were two-sided, and p < 0.05 was considered statistically significant.

## 3 Results

### 3.1 Study population and baseline characteristics

The final study population included 468 patients with CRC (nmCRC, n = 391; mCRC, n = 77), and the inclusion/exclusion process and cohort construction are summarized in **Figure 1**. Of the 468 included patients, 256 (54.7%) were from Center 1 and 212 (45.3%) were from Center 2. Baseline characteristics stratified by study center are provided in **Table 1**. Most baseline variables were broadly comparable between centers, although significant between-center differences were observed for IMATI (p = 0.014) and CEA distribution (p = 0.008). Among the surgical nmCRC cohort (n = 391), 96 patients (24.6%) developed distant metastasis during follow-up. Compared with nmCRC (**Table 2**), mCRC had higher SATI, VATI, and VSR (all p ≤0.002), whereas IMATI did not differ significantly (p = 0.650). The proportions of elevated CEA and CA19–9 were higher in mCRC (p = 0.025 and p < 0.001, respectively). The proportion of BMI ≥ 25 kg/m^2^ was higher in mCRC but did not reach statistical significance (p = 0.073).

**Table 1:** Baseline characteristics stratified by study center.

Variables	Overall (n=468)	Center 1 (n=256)	Center 2 (n=212)	P value
Age	66.00 (56.00–73.00)	67.00 (56.00–73.25)	64.00 (55.00–72.00)	0.412
IMATI	9.98 (7.39–13.53)	9.36 (7.10–12.80)	10.44 (7.98–14.30)	0.014
SATI	46.13 (36.27–63.96)	48.70 (36.31–64.67)	43.65 (35.89–62.03)	0.408
VATI	41.10 (32.84–49.57)	41.09 (32.81–48.22)	41.11 (33.16–51.56)	0.632
VSR	0.86 (0.61–1.24)	0.83 (0.58–1.24)	0.90 (0.63–1.24)	0.33
nmCRC/mCRC, n (%)				0.779
nmCRC	391 (83.5)	215 (84.0)	176 (83.0)	
mCRC	77 (16.5)	41 (16.0)	36 (17.0)	
Sex				0.488
Male	229 (48.9)	129 (50.4)	100 (47.2)	
Female	239 (51.1)	127 (49.6)	112 (52.8)	
CEA, n (%)				0.008
Normal	267 (57.1)	132 (51.6)	135 (63.7)	
Abnormal	201 (42.9)	124 (48.4)	77 (36.3)	
CA19-9, n (%)				0.168
Normal	350 (74.8)	185 (72.3)	165 (77.8)	
Abnormal	118 (25.2)	71 (27.7)	47 (22.2)	
BMI, n (%)				0.449
<25 kg/m^2	337 (72.0)	188 (73.4)	149 (70.3)	
≥ 25 kg/m^2	131 (28.0)	68 (26.6)	63 (29.7)	
Tumor location, n (%)				0.382
Colon	317 (67.7)	169 (66.0)	148 (69.8)	
Rectum	151 (32.3)	87 (34.0)	64 (30.2)	

Data are presented as count (%) for categorical variables and median (Q1, Q3) for continuous variables. nmCRC, non-metastatic colorectal cancer; mCRC, metastatic colorectal cancer; BMI, body mass index; CA19-9, carbohydrate antigen 19-9; CEA, carcinoembryonic antigen; SATI, Subcutaneous adipose tissue index; VATI, Visceral adipose tissue index; IMATI, Intermuscular adipose tissue index; VSR, Visceral-to-subcutaneous fat area ratio.

**Table 2:** Baseline characteristics of the overall colorectal cancer cohort.

Variables	Total(N = 468)	nmCRC(N = 391)	mCRC(N = 77)	P value
SATI	46.13 (36.27–63.96)	44.43 (34.77–62.01)	53.35 (42.16–75.75)	<0.001
IMATI	9.98 (7.39–13.53)	9.98 (7.26–13.69)	10.35 (8.25–12.90)	0.650
VATI	41.10 (32.84–49.57)	38.05 (32.01–46.14)	58.82 (48.20–63.11)	<0.001
VSR	0.86 (0.61–1.24)	0.83 (0.59–1.20)	1.09 (0.68–1.29)	0.002
Age (years)	66.00 (56.00–73.00)	66.00 (56.00–73.00)	60.00 (55.00–73.00)	0.117
Sex, n (%)				0.243
Men	229 (48.9)	196 (50.1)	33 (42.9)	
Women	239 (51.1)	195 (49.9)	44 (57.1)	
CEA, n (%)				0.025
Normal	267 (57.1)	232 (59.3)	35 (45.5)	
Abnormal	201 (42.9)	159 (40.7)	42 (54.5)	
CA19-9, n (%)				<0.001
Normal	350 (74.8)	306 (78.3)	44 (57.1)	
Abnormal	118 (25.2)	85 (21.7)	33 (42.9)	
BMI, n (%)				0.073
<25 kg/m^2	337 (72.0)	288 (73.7)	49 (63.6)	
≥ 25 kg/m^2	131 (28.0)	103 (26.3)	28 (36.4)	
Tumor location, n (%)				0.758
Colon	317 (67.7)	266 (68.0)	51 (66.2)	
Rectum	151 (32.3)	125 (32.0)	26 (33.8)	
Study center, n (%)				0.779
Center 1	256 (54.7)	215 (55.0)	41 (53.2)	
Center 2	212 (45.3)	176 (45.0)	36 (46.8)	

Data are presented as count (%) for categorical variables and median (Q1, Q3) for continuous variables. nmCRC, non-metastatic colorectal cancer; mCRC, metastatic colorectal cancer; BMI, body mass index; CA19-9, carbohydrate antigen 19-9; CEA, carcinoembryonic antigen; LVI, lymphovascular invasion; PNI, perineural invasion; SATI, Subcutaneous adipose tissue index; VATI, Visceral adipose tissue index; IMATI, Intermuscular adipose tissue index; VSR, Visceral-to-subcutaneous fat area ratio.

### 3.2 Body composition differences and factors associated with synchronous metastasis

After 1:1 matching for age, sex, CEA, CA19-9, study center, and tumor location, 77 matched pairs of mCRC and nmCRC were obtained. Balance diagnostics indicated acceptable post-matching covariate balance, with all assessed variables showing absolute SMDs < 0.10 (**Table 3**). In the matched cohort, the proportions of elevated CEA and CA19–9 were comparable between groups (p = 0.746 and p = 0.623, respectively), and BMI categories did not differ significantly (p = 0.392). Regarding body composition, mCRC showed higher SATI (p = 0.015), markedly higher VATI (p < 0.001), and higher VSR (p = 0.003), whereas IMATI did not differ (p = 0.826) (**Table 3**). In the multivariable logistic regression model, VATI remained independently associated with synchronous metastasis (**Table 4**). Specifically, each 1-unit increase in VATI was associated with higher odds of synchronous metastasis (OR, 1.110; 95% CI, 1.067–1.155; p < 0.001). When VATI was standardized, the association remained robust, with per IQR increase associated with significantly higher odds of synchronous metastasis (OR, 10.545; 95% CI, 4.281–25.974; p < 0.001). Additional adjustment for the matched variables yielded consistent results (OR = 1.131, 95% CI: 1.070–1.195, P<0.001), supporting the robustness of the association between VATI and synchronous metastasis.

**Table 3:** Comparison of clinical characteristics and body composition parameters between nmCRC and mCRC after 1:1 matching for age, sex, CEA, CA19-9, study center, and tumor location.

Variable	Overall (n=154)	nmCRC (n=77)	mCRC (n=77)	P value	SMD
VATI	48.09 (37.53–60.09)	39.29 (31.63–47.10)	58.82 (48.20–63.11)	<0.001	–
Sex, n (%)				0.746	0.052
Male	68 (44.2)	35 (45.5)	33 (42.9)		
Female	86 (55.8)	42 (54.5)	44 (57.1)		
Age (years)	60.00 (54.00–73.00)	60.00 (52.00–73.00)	60.00 (55.00–73.00)	0.697	0.077
CEA, n (%)				0.746	0.052
Normal	68 (44.2)	33 (42.9)	35 (45.5)		
Abnormal	86 (55.8)	44 (57.1)	42 (54.5)		
CA19-9, n (%)				0.623	0.079
Normal	91 (59.1)	47 (61.0)	44 (57.1)		
Abnormal	63 (40.9)	30 (39.0)	33 (42.9)		
Tumor location, n (%)				0.614	0.081
Colon	99 (64.3)	48 (62.3)	51 (66.2)		
Rectum	55 (35.7)	29 (37.7)	26 (33.8)		
Study center, n (%)				0.747	0.052
Center 1	80 (51.9)	39 (50.6)	41 (53.2)		
Center 2	74 (48.1)	38 (49.4)	36 (46.8)		
IMATI	10.15 (7.71–13.78)	10.11 (6.68–14.38)	10.35 (8.25–12.90)	0.826	–
SATI	50.78 (39.17–70.25)	47.83 (36.31–65.79)	53.35 (42.16–75.75)	0.015	–
VSR	0.92 (0.63–1.24)	0.86 (0.55–1.09)	1.09 (0.68–1.29)	0.003	–
BMI				0.392	–
<25 kg/m^2^	103 (66.9)	54 (70.1)	49 (63.6)		
≥ 25 kg/m^2^	51 (33.1)	23 (29.9)	28 (36.4)		

Data are presented as count (%) for categorical variables and median (Q1, Q3) for continuous variables. nmCRC, non-metastatic colorectal cancer; mCRC, metastatic colorectal cancer; BMI, body mass index; CA19-9, carbohydrate antigen 19-9; CEA, carcinoembryonic antigen; LVI, lymphovascular invasion; PNI, perineural invasion; SATI, Subcutaneous adipose tissue index; VATI, Visceral adipose tissue index; IMATI, Intermuscular adipose tissue index; VSR, Visceral-to-subcutaneous fat area ratio.

**Table 4:** Multivariable logistic regression analysis of factors associated with synchronous metastasis in colorectal cancer in the matched cohort.

Variables	OR (95%CI)	P value
SATI	1.005 (0.986–1.025)	0.612
VATI	1.110 (1.067–1.155)	<0.001
VSR	1.010 (0.387–2.633)	0.984

OR, odds ratio; CI, confidence interval; SATI, Subcutaneous adipose tissue index; VATI, Visceral adipose tissue index; VSR, Visceral-to-subcutaneous fat area ratio.

### 3.3 Early versus late postoperative metastasis in the nmCRC cohort

Among patients who developed postoperative distant metastasis (n = 96), 76 were classified as early metastasis (≤ 2 years after surgery) and 20 as late metastasis (> 2 years). In this exploratory descriptive comparison, body composition, clinical, and pathologic variables were compared directly between the two groups (**Table 5**). No statistically significant differences in VATI, SATI, IMATI, VSR, or BMI were observed between the early and late metastasis groups. Likewise, no statistically significant differences were observed in most clinical and pathologic variables, although grade and CEA showed between-group differences in the univariable comparison.

**Table 5:** Baseline characteristics of nmCRC patients with postoperative metastasis stratified by time to metastasis (2-year cutoff).

Variables	Total(N = 96)	Early metastasis(N = 76)	Late metastasis(N = 20)	P value
Age (years)	66.00(55.00,72.00)	67.00(55.75, 70.00)	63.00(53.75, 76.00)	0.882
Grade, n (%)				0.017
G1	30 (31.2%)	29(38.2%)	1(5.0%)	
G2	53 (55.2%)	38(50.0%)	15(75.0%)	
G3	13 (13.5%)	9(11.8%)	4(20.0%)	
IMATI	10.23(7.43,14.33)	10.29(8.16, 14.33)	10.17(6.67, 14.33)	0.424
SATI	45.89(39.17,62.01)	43.53(39.17, 60.77)	53.11(41.47, 71.48)	0.419
VATI	43.05(33.17,47.10)	43.05(33.28, 47.37)	40.39(32.84, 43.99)	0.259
VSR	0.74(0.52,1.20)	0.74(0.60, 1.14)	0.74(0.52, 1.20)	0.762
Tumor location, n (%)				0.722
Colon	64 (66.7%)	50 (65.8%)	14 (70.0%)	
Rectum	32 (33.3%)	26 (34.2%)	6 (30.0%)	
Sex, n (%)				0.691
Men	47 (49.0%)	38 (50.0%)	9 (45.0%)	
Women	49 (51.0%)	38 (50.0%)	11 (55.0%)	
T, n (%)				0.508
1-2	3 (3.1%)	2 (2.6%)	1 (5.0%)	
3-4	93 (96.9%)	74 (97.4%)	19 (95.0%)	
N, n (%)				0.082
N0	24 (25.0%)	16 (21.1%)	8 (40.0%)	
1-2	72 (75.0%)	60 (78.9%)	12 (60.0%)	
CEA, n (%)				0.029
Normal	33 (34.4%)	22 (28.9%)	11 (55.0%)	
Elevated	63 (65.6%)	54 (71.1%)	9 (45.0%)	
CA19-9, n (%)				0.089
Normal	56 (58.3%)	41 (53.9%)	15 (75.0%)	
Elevated	40 (41.7%)	35 (46.1%)	5 (25.0%)	
PNI, n (%)				0.284
No	38 (39.6%)	28 (36.8%)	10 (50.0%)	
Yes	58 (60.4%)	48 (63.2%)	10 (50.0%)	
LVI, n (%)				0.433
No	31 (32.3%)	26 (34.2%)	5 (25.0%)	
Yes	65 (67.7%)	50 (65.8%)	15 (75.0%)	
BMI, n (%)				0.684
<25 kg/m^2^	66 (68.8%)	53 (69.7%)	13 (65.0%)	
≥ 25 kg/m^2^	30 (31.2%)	23 (30.3%)	7 (35.0%)	

Data are presented as count (%) for categorical variables and median (Q1, Q3) for continuous variables. BMI, body mass index; CA19-9, carbohydrate antigen 19-9; CEA, carcinoembryonic antigen; LVI, lymphovascular invasion; PNI, perineural invasion; SATI, Subcutaneous adipose tissue index; VATI, Visceral adipose tissue index; IMATI, Intermuscular adipose tissue index; VSR, Visceral-to-subcutaneous fat area ratio.

### 3.4 Metastasis-free survival stratified by sex-specific median VATI

**Figure 3:**
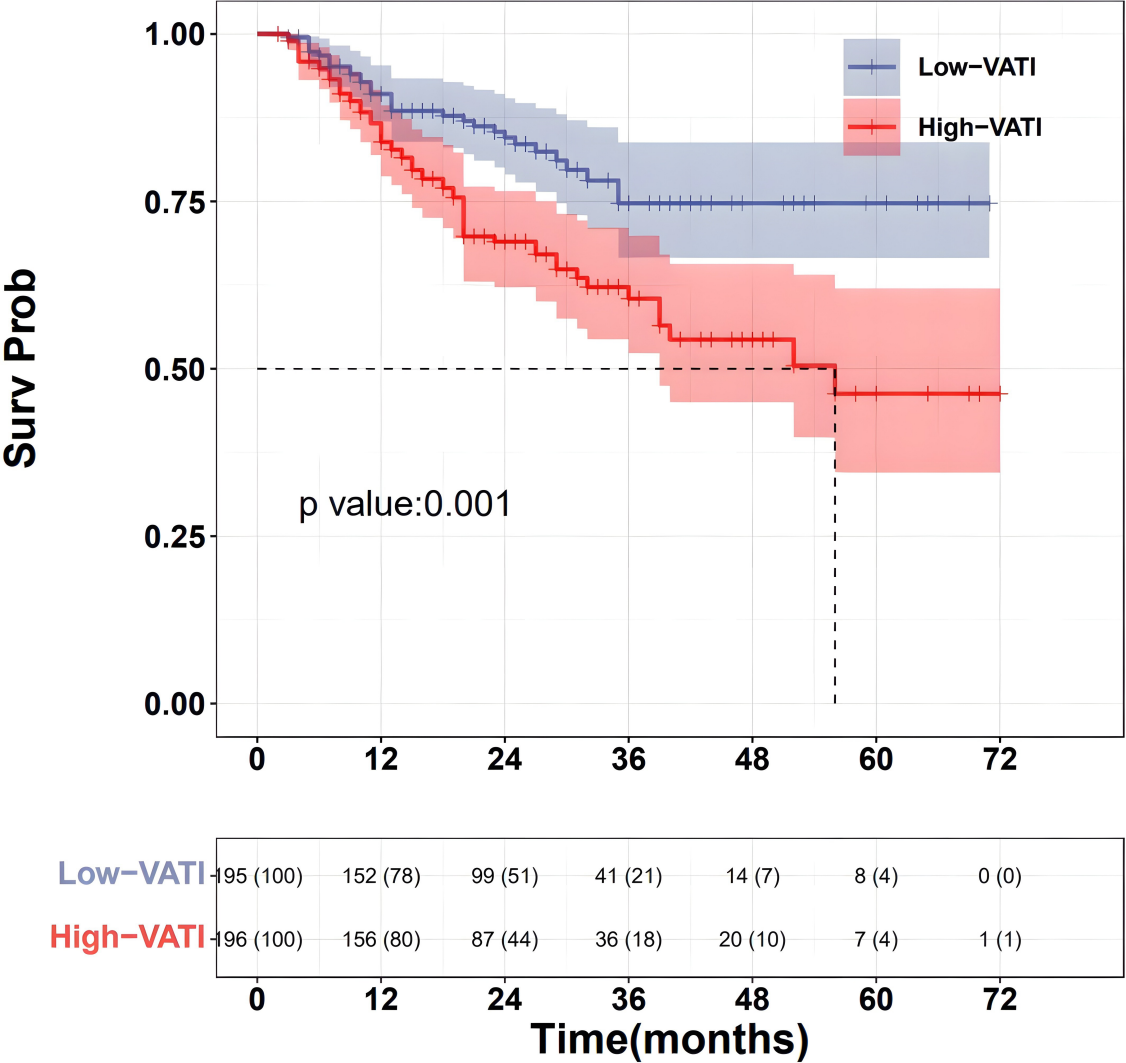
Kaplan-Meier curves for MFS stratified by sex-specific median VATI (low VATI, n = 195; high VATI, n = 196). MFS, metastasis-free survival; VATI, visceral adipose tissue index.

The median follow-up duration for the surgical nmCRC cohort (n = 391) was 22.0 months (IQR, 12.0–31.0 months). Patients were stratified into low-VATI (n = 195) and high-VATI (n = 196) groups using sex-specific median VATI values. MFS differed significantly between groups (log-rank p = 0.001) (**Figure 3**). On univariable Cox analysis, CA19-9, CEA, grade, IMATI, T stage, N stage, PNI, LVI, and VATI were associated with MFS (**Table 6**). On multivariable Cox analysis, VATI remained independently associated with shorter MFS (HR, 1.017; 95% CI, 1.003-1.032; p = 0.021) (**Table 6**), when VATI was additionally expressed per one IQR increase, each one-IQR increase in VATI was associated with a significantly higher hazard of postoperative metastasis (HR, 1.269; 95% CI, 1.033–1.559; p = 0.023). Elevated CA19-9 (HR, 2.074; 95% CI, 1.324-3.249; p = 0.001), elevated CEA (HR, 2.121; 95% CI, 1.329-3.385; p = 0.002), N stage (HR, 2.568; 95% CI, 1.549-4.257; p < 0.001), and LVI (HR, 2.499; 95% CI, 1.557-4.010; p < 0.001) were also independent adverse factors. In univariable Cox analysis, study center was not significantly associated with MFS (HR, 0.783; 95% CI, 0.520–1.180; p = 0.242). However, in a sensitivity Cox model with study center forced into the multivariable analysis, the association between VATI and shorter MFS remained materially unchanged (HR per IQR increase, 1.262; 95% CI, 1.029–1.549; p = 0.026). The proportional hazards assumption was assessed using Schoenfeld residuals. No significant violation of the proportional hazards assumption was observed for the final multivariable Cox model globally (χ² = 7.198, df = 9, p = 0.616) or for VATI specifically (χ² = 0.004, p = 0.951).

**Table 6:** Univariable and multivariable Cox proportional hazards regression for MFS in the nmCRC cohort.

Variables	Crude HR (95%CI)	uni-P value	Adj HR (95%CI)	multi-P value
BMI (≥ 25 kg/m^2^ vs <25 kg/m^2^)	1.358(0.882,2.091)	0.165		
CA19-9 (≥ 37 U/mL)	3.414(2.266,5.143)	<0.001	2.074(1.324,3.249)	0.001
CEA (≥ 5 ng/mL)	3.527(2.312,5.380)	<0.001	2.121(1.329,3.385)	0.002
Grade	0.636(0.460,0.879)	0.006	0.815(0.586,1.134)	0.224
IMATI (per 1 cm^2^/m^2^)	1.048(1.003,1.095)	0.037	1.047(0.999,1.096)	0.053
N stage (N1–2 vs N0)	4.673(2.941,7.425)	<0.001	2.568(1.549,4.257)	<0.001
PNI (yes)	2.868(1.895,4.341)	<0.001	1.167(0.733,1.858)	0.516
SATI (per 1 cm^2^/m^2^)	1.006(0.997,1.014)	0.177		
Sex(men)	1.065(0.714,1.590)	0.756		
T stage (T3–4 vs T1-2)	5.503(1.742,17.379)	0.004	2.564(0.783,8.396)	0.120
Tumor location (rectum vs colon)	1.223(0.800,1.870)	0.353		
VATI (per 1 cm^2^/m^2^)	1.028(1.013,1.043)	<0.001	1.017(1.003,1.032)	0.021
LVI (yes)	3.960(2.574,6.092)	<0.001	2.499(1.557,4.010)	<0.001
VSR (per 1 unit)	0.753(0.477,1.190)	0.225		
Age (years)	1.001(0.984,1.018)	0.925		
Study center (center 1 vs center 2)	0.783(0.520,1.180)	0.242		

BMI, body mass index; CA19-9, carbohydrate antigen 19-9; CEA, carcinoembryonic antigen; LVI, lymphovascular invasion; PNI, perineural invasion; SATI, Subcutaneous adipose tissue index; VATI, Visceral adipose tissue index; IMATI, Intermuscular adipose tissue index; VSR, Visceral-to-subcutaneous fat area ratio; HR, Hazard Ratio; CI, confidence interval; MFS, metastasis-free survival.

### 3.5 Interobserver reproducibility of body composition measurement

In the subset of 50 cases, interobserver agreement was excellent for VATI (ICC, 0.995; 95% CI, 0.991–0.997), SATI (ICC, 0.996; 95% CI, 0.993–0.998), and IMATI (ICC, 0.988; 95% CI, 0.979–0.993).

## 4 Discussion

In this two-center retrospective cohort study, higher preoperative VATI was independently associated with synchronous metastasis at diagnosis and with shorter MFS after curative-intent surgery among patients with nmCRC. These findings suggest that VATI may represent a host-related metabolic phenotype associated with metastatic risk and may provide complementary information beyond established clinicopathologic factors. In the exploratory comparison among patients who developed postoperative metastasis, no statistically significant differences in body composition metrics were observed between the early and late metastasis groups. In the nmCRC cohort, traditional tumor-related factors including elevated CA19–9 and CEA, nodal involvement, and LVI were also independently associated with shorter MFS, highlighting the complementary roles of host metabolic phenotype and tumor aggressiveness. Although traditional postoperative pathological factors such as LVI are associated with the risk of metastasis (20), they need to be obtained after the operation, while VATI can provide supplementary risk information before the operation.

Compared with BMI, VATI more directly captures inter-individual differences in visceral fat burden and related metabolic heterogeneity. Patients with similar BMI can have markedly different VAT accumulation, which may dilute associations between BMI and oncologic outcomes. Prior studies have linked CT-derived body composition parameters to CRC recurrence and survival (21), and associations between higher VATI and adverse outcomes have been reported in clinical cohorts (7, 22). Our findings extend this evidence by demonstrating that VATI retained an independent association with synchronous metastasis and postoperative MFS after matching for key baseline covariates. Given that VATI can be obtained opportunistically from routine staging CT without additional testing, it may represent a cost-effective biomarker for clinical implementation. In addition, formal interobserver analysis demonstrated excellent reproducibility of VATI measurement, supporting the technical robustness of this CT-derived biomarker. Importantly, the association between VATI and shorter MFS remained materially unchanged in a sensitivity Cox model with study center forced into the multivariable analysis, suggesting that the observed relationship was not solely driven by center-related heterogeneity.

Mechanistically, VAT accumulation is commonly accompanied by insulin resistance, adipokine dysregulation, and chronic low-grade inflammation. Adipocytes and adipose stromal cells, as well as their exosomes, can remodel the tumor microenvironment via cytokines and growth factors such as interleukin-6 and hepatocyte growth factor and activate pro-metastatic signaling pathways, thereby promoting invasion, dissemination, and treatment resistance (14, 15). From the perspective of metabolism, the interaction between fat and tumor is considered an important background for tumor progression (23). Imaging and deep learning studies also suggest that visceral fat phenotypes may influence the risk of occult peritoneal metastasis in CRC (9, 24). From a clinical perspective, a higher VATI may indicate a metastatic-prone host milieu, supporting more vigilant assessment for peritoneal and distant metastases at baseline and potentially more intensive surveillance after surgery.

This study has limitations. First, the retrospective observational design introduces the possibility of residual confounding and selection bias despite matching and multivariable adjustment. For instance, it was not possible to obtain the postoperative T stage, N stage and tumor grade of patients with synchronous metastatic colorectal cancer. Second, VATI primarily reflects the quantity of visceral fat and does not capture fat quality metrics such as adipose tissue radiodensity, which has been associated with CRC survival (25). Third, the exploratory early versus late metastasis analysis was descriptive in nature, and the late-metastasis subgroup was relatively small. Future prospective multicenter studies are warranted to validate thresholds, incorporate additional body composition and treatment variables, and evaluate integrated models that combine tumor imaging features with body composition metrics.

In conclusion, CT-derived VATI at the L3 level was independently associated with synchronous metastasis at diagnosis and with shorter MFS after curative-intent surgery in nmCRC. Preoperative VATI may serve as a reproducible opportunistic imaging biomarker to refine metastatic risk stratification and postoperative surveillance strategies in CRC, pending prospective external validation.

## Data Availability

The data analyzed in this study is subject to the following licenses/restrictions: The datasets generated and/or analyzed during the current study are available from the corresponding author on reasonable request. Requests to access these datasets should be directed to guojinjun1972@163.com.
